# Impact of Tricuspid Valve Repair on Less Than Moderate Tricuspid Regurgitation After Degenerative Mitral Repair

**DOI:** 10.1016/j.jacasi.2025.01.012

**Published:** 2025-03-25

**Authors:** Jinren Du, Yichen Zhao, Kemin Liu, Qing Ye, Cheng Zhao, Jie Han, Xu Meng, Fei Meng, Tiange Luo, Baiyu Tian, Jiangang Wang

**Affiliations:** Department of Cardiac Surgery, Beijing Anzhen Hospital, Capital Medical University, Beijing, China

**Keywords:** atrial fibrillation, mitral valve insufficiency, tricuspid valve insufficiency

## Abstract

**Background:**

Whether tricuspid valve (TV) repair should be performed during degenerative mitral valve repair in patients with less than moderate regurgitation remains controversial.

**Objectives:**

This study aimed to explore the clinical outcomes of degenerative mitral valve repair for less than moderate tricuspid regurgitation (TR).

**Methods:**

Between 2010 and 2019, 541 patients with less than moderate TR underwent mitral valve repair for degenerative mitral disease at the Beijing Anzhen Hospital. Among these patients, 255 underwent concomitant TV repair. The median follow-up was 8 years (Q1-Q3: 6-11 years). The primary endpoint was the development of moderate TR. The secondary endpoint was death, recurrent mitral regurgitation (MR), reoperation for MR or TR, new-onset atrial fibrillation, and permanent pacemaker implantation. Propensity score matching was performed to reduce selection bias.

**Results:**

After baseline adjustment, propensity score matching analysis identified 207 pairs. There were no significant differences in the primary endpoints between the patients who underwent simultaneous TV repair and those who did not. Mortality, recurrent MR, permanent pacemaker implantation, and reoperation for MR or TR did not differ significantly between groups. Concomitant TV repair only contributed to new-onset atrial fibrillation, with 24 (11.6%) in the TV-repair group and 7 (3.4%) in the no-repair group (*P =* 0.001).

**Conclusions:**

Considering its minimal effects on the development of TR and potential contributions to postoperative new-onset atrial fibrillation during follow-up among these patients, more aggressive TV repair is not encouraged among patients with less than moderate TR during MV surgery.

Degenerative mitral regurgitation (MR) is a common public health problem.^1^ In China, caused by rapid economic development and the aging population, the prevalence of degenerative valvular heart disease has increased.^2^ Tricuspid regurgitation (TR) combined with degenerative MR is common.^3^ Severe TR does not improve after isolated mitral valve surgery, usually requiring concomitant tricuspid valve (TV) repair. Current practical guidelines offer Class I recommendations for simultaneous TV repair in patients with severe TR.^4^

However, the need for TV repair in moderate and less than moderate TR remains controversial. Supporters of TV repair show that without concomitant TV repair, several patients will have TR progression. TV repair may prevent the development of TR and right ventricle dysfunction.^5^, ^6^, ^7^, ^8^, ^9^ Opponents have shown that TV regurgitation is unlikely to progress after mitral valve repair.^3^^,^^10^ Additionally, concomitant TV repair may increase the risk of adverse events and does not seem to have sufficient clinical benefits.^11^^,^^12^ Therefore, clarifying whether TV repair should be performed during mitral valve repair in patients with less than moderate TR is essential.

To date, no such relevant studies have been conducted in China. In this study, we retrospectively analyzed patients with less than moderate TR who underwent mitral valve repair caused by degenerative mitral disease. We compared the development of moderate TR, mortality, recurrent MR, new-onset atrial fibrillation, permanent pacemaker (PPM) implantation, and reoperation caused by MR or TR between patients who underwent simultaneous TV repair and those who did not.

## Methods

### Study patients and outcome measures

At our institution, 754 consecutive patients with less than moderate TR underwent mitral valve repair for degenerative mitral disease between January 2010 and December 2019. The exclusion criteria were atrial fibrillation, congenital heart disease, age <18 years, and history of other concomitant heart surgeries. After excluding 77 patients with congenital heart disease and a history of other concomitant heart surgeries and 136 patients with preoperative atrial fibrillation, a total of 541 patients were enrolled in this study, including 255 who underwent simultaneous TV repair and 286 who did not. This study was approved by the Ethics Committee of Beijing Anzhen Hospital(approval number: 20242002X). Before enrollment in the study, the patients received oral and written information about the study and provided written informed consent.

Transthoracic echocardiogram and electrocardiogram (ECG) were performed at 6, 12, and 24 months. After 24 months, follow-up transthoracic echocardiogram and ECG were performed annually. The primary endpoint was the development of moderate TR. The secondary endpoints were new-onset onset atrial fibrillation, death, recurrent MR, PPM implantation, and heart reoperation caused by MR or TR. The degrees of TR and MR (mild, moderate, or severe) were determined through a combination of qualitative and quantitative methods. In terms of quantitative methods, we termed an effective regurgitant orifice area <0.2 cm^2^ as mild, 0.2 to 0.39 cm^2^ as moderate, and ≥0.4 cm^2^ as severe.^13^ Recurrent MR was defined as moderate MR.

### Surgery

All surgeries were performed under general anesthesia with tracheal intubation. Transesophageal echocardiography was performed preoperatively to examine the mitral valve pathology. The choice of mitral valve repair technique was based on the surgeon's preference, experience, and practice. All patients underwent tricuspid valvuloplasty with dilation of the tricuspid annulus (≥40 mm). TV annuloplasty was performed for TV repair. The sizing of the tricuspid ring was based on the combined surface area of the posterior and anterior tricuspid leaflets, which extended via a right-angle hook. The ring was implanted using simple, interrupted mattress sutures by sparing the septal annulus and conduction tissue in the region of the apex of the triangle of Koch. Four surgeons with extensive experience in mitral and TV surgeries at our center performed the procedures mentioned in this study.

### Statistical analyses

Continuous variables are expressed as mean ± SD or median (Q1-Q3), and categorical variables are reported as frequencies and percentages. Differences between groups were analyzed using Student's *t*-test or Mann-Whitney *U* test for continuous variables and the chi-square or Fisher exact test for categorical variables. The significance level for all statistical tests was bilateral (*P <* 0.05). Kaplan-Meier analysis was used to evaluate long-term survival, MR recurrence, and reoperation rates, and the log-rank method was used to compare differences between survival curves. Cox proportional hazards regression models were used to analyze the incidence of new-onset atrial fibrillation from any cause. The proportional hazards assumption in the Cox models was tested with Schoenfeld residuals. Propensity score matching was performed to reduce potentially confounding factors. Logistic regression models were used to calculate propensity scores. Cases from the TV-repair group were matched 1:1 with cases from the no TV-repair group, using a caliper of 0.1 of the SD of the logit of the propensity score without replacement. All reported *P* values were 2-tailed. SPSS Statistics version 26.0 (IBM Corporation) and R statistical software version 4.4.1 (R Foundation for Statistical Computing) were used for the statistical analyses.

## Results

### Baseline characteristics

Among the 541 patients in this study, 255 underwent simultaneous TV repair. The remaining 286 patients did not undergo TV repair. During the follow-up period, 43 patients were lost to follow-up and 498 patients were tracked. The median age of the study population was 52 (17) years, with 24.6% (133 of 541) ≥60 years of age. Before matching, TV repair patients were older *(P =* 0.009) and had a larger left atrial diameter *(P =* 0.047), left ventricular end-systolic diameter *(P =* 0.008), and left ventricular end-diastolic diameter *(P =* 0.001). After matching, 207 pairs of patients were analyzed, and most variables were well-balanced. The baseline characteristics of the patients are summarized in Table 1.

**Table 1:** Patient Baseline Characteristics

	Unmatched			Matched		
	No Repair (n = 286)	TV Repair (n = 255)	*P* Value	No Repair (n = 207)	TV Repair (n = 207)	*P* Value
Age, y	49 (20)	53 (15)	0.009	52 (19)	53 (15)	0.633
Height, cm	167.8 ± 8.2	168.4 ± 8.2	0.456	168.3 ± 8.2	168.4 ± 8.3	0.933
Weight, kg	70.0 ± 12.3	70.0 ± 12.0	0.901	70.2 ± 12.1	70.1 ± 12.4	0.950
Female	90 (31.5)	78 (30.6)	0.825	65 (31.4)	64 (30.9)	0.915
Smoking	89 (31.1)	81 (31.8)	0.872	65 (31.4)	67 (32.4)	0.833
HF	3 (1.0)	0 (0)	0.289	0 (0)	0 (0)	1.000
CAD	7 (2.4)	7 (2.7)	0.828	6 (2.9)	6 (2.9)	1.000
Hypertension	101 (35.3)	102 (40.0)	0.261	77 (37.2)	82 (39.6)	0.613
Diabetes mellitus	13 (4.5)	9 (3.5)	0.550	4 (1.9)	7 (3.4)	0.359
Dyslipidemia	3 (1.0)	5 (2.0)	0.603	3 (1.4)	5 (2.4)	0.721
Stroke	8 (2.8)	11 (4.3)	0.339	8 (3.9)	8 (3.9)	1.000
NYHA functional class						
I	14 (4.9)	11 (4.3)	0.792	10 (4.8)	11 (5.3)	0.641
II	224 (78.3)	200 (78.4)		160 (77.3)	154 (74.4)	
III	46 (16.1)	42 (16.5)		36 (17.4)	40 (19.3)	
IV	2 (0.7)	2 (0.8)		1 (0.5)	2 (1.0)	
COPD	3 (1.0)	2 (0.8)	1.000	1 (0.5)	2 (1.0)	1.000
ESRD	1 (0.3)	2 (0.8)	0.921	1 (0.5)	1 (0.5)	1.000
LAD, mm	43.5 ± 7.8	44.8 ± 8.2	0.047	44.4 ± 7.7	44.7 ± 8.9	0.623
LVEF, %	64.4 ± 6.0	63.7 ± 6.1	0.217	64.0 ± 6.2	64.2 ± 5.7	0.745
LVEDD, mm	55.9 ± 6.5	57.9 ± 6.4	0.001	57.0 ± 6.3	57.1 ± 6.3	0.910
LVESD, mm	35.7 ± 5.5	37.0 ± 5.6	0.008	36.4 ± 5.4	36.4 ± 5.3	0.961
CPB time, min	93 (52)	88 (27)	0.103	92 (49)	88 (25)	0.252
Clamping time, min	65 (39)	61 (23)	0.228	64 (38)	61 (23)	0.496

Values are n (%) or mean ± SD.

CAD = coronary artery disease; COPD = chronic obstructive pulmonary disease; CPB = cardiopulmonary bypass; ESRD = end-stage renal disease; HF = heart failure; LAD = left atrial diameter; LVEDD = left ventricular end-diastolic dimension; LVEF = left ventricular ejection fraction; LVESD = left ventricular end-systolic dimension; TV = tricuspid valve.

### Mortality and development of moderate TR

**Figure 1:**
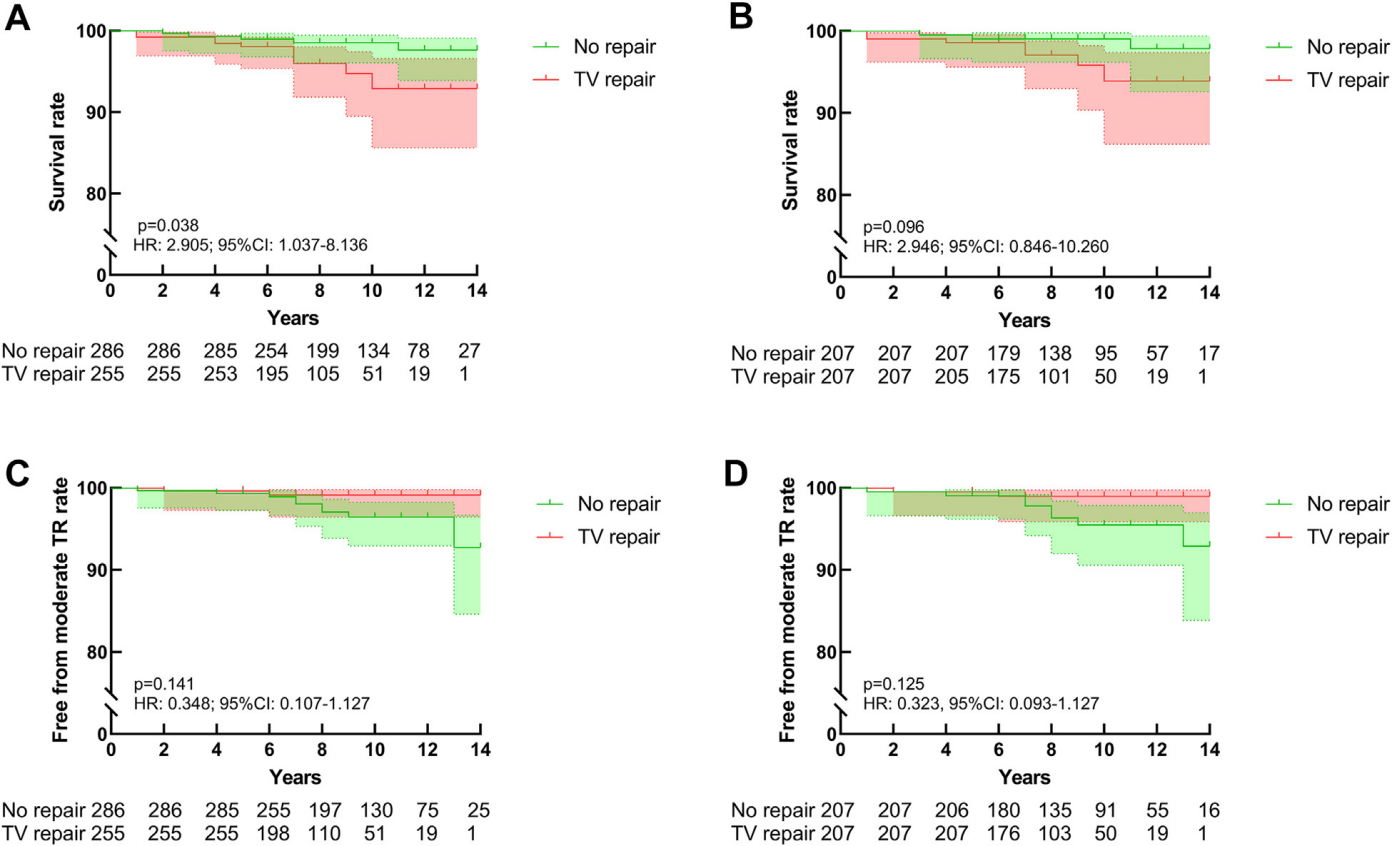
Kaplan-Meier Curves for Survival and the Primary Endpoint (A and B) Cumulative survival rates before and after matching. (C and D) Cumulative rates of freedom from the development of moderate tricuspid regurgitation (TR) before and after matching. TV = tricuspid valve.

The clinical outcomes are summarized in Table 2. During the 8 years (Q1-Q3: 6-11 years) of follow-up, death occurred in 5 of 286 patients (1.7%) in the group that did not receive TV repair and in 10 of 255 (3.9%) in the group that did. After propensity score matching, the number of deaths was reduced to 3 of 207 patients (1.4%) in the group without TV repair and 7 of 207 (3.3%) in the TV-repair group. The survival rate did not significantly differ between the 2 groups after matching (HR: 2.946; 95% CI: 0.846-10.260; *P =* 0.096). A total of 12 patients presented with moderate degrees of TR, 2 of whom underwent TR repair and 10 did not. The rate of moderate TR development did not differ significantly between the 2 groups (HR: 0.348; 95% CI: 0.107-1.127; *P =* 0.141). After matching, there was still no significant difference in the rate of development of moderate TR between the 2 groups (HR: 0.323; 95% CI: 0.093-1.127; *P =* 0.125). Figure 1 depicts the Kaplan-Meier plots for the cumulative survival rates and freedom from moderate TR development rate before and after matching.

**Table 2:** Clinical Outcomes Before and After Matching

	Before Matching					After Matching				
	No Repair (n = 286)	TV Repair (n = 255)	*P* Value	HR	95% CI	No Repair (n = 207)	TV Repair (n = 207)	*P* Value	HR	95% CI
Death	5 (1.7)	10 (3.9)	0.038	2.905	1.037-8.136	3 (1.4)	7 (3.3)	0.096	2.946	0.846-10.260
Development of moderate TR	10 (3.5)	2 (0.8)	0.141	0.348	0.107-1.127	8 (3.9)	2 (1.0)	0.125	0.323	0.093-1.127
Reoperation	6 (2.1)	3 (1.2)	0.535	0.652	0.174-2.434	5 (2.4)	3 (1.4)	0.488	0.606	0.152-2.425
Recurrent MR	22 (7.7)	13 (5.1)	0.785	0.912	0.463-1.796	19 (9.2)	13 (6.3)	0.675	0.862	0.429-1.732
PPM implantation	8 (2.8)	5 (2.0)	0.120	2.335	0.772-7.063	3 (1.4)	5 (2.4)	0.381	1.874	0.468-7.513
New-onset atrial fibrillation	8 (2.8)	31 (12.2)	0.001	2.893	1.528-5.477	7 (3.4)	24 (11.6)	0.001	3.906	1.929-7.909

Values are n (%) unless otherwise indicated.

MR = recurrent mitral regurgitation; PPM = permanent pacemaker; TR = tricuspid regurgitation.

### Reoperation and recurrent MR

**Figure 2:**
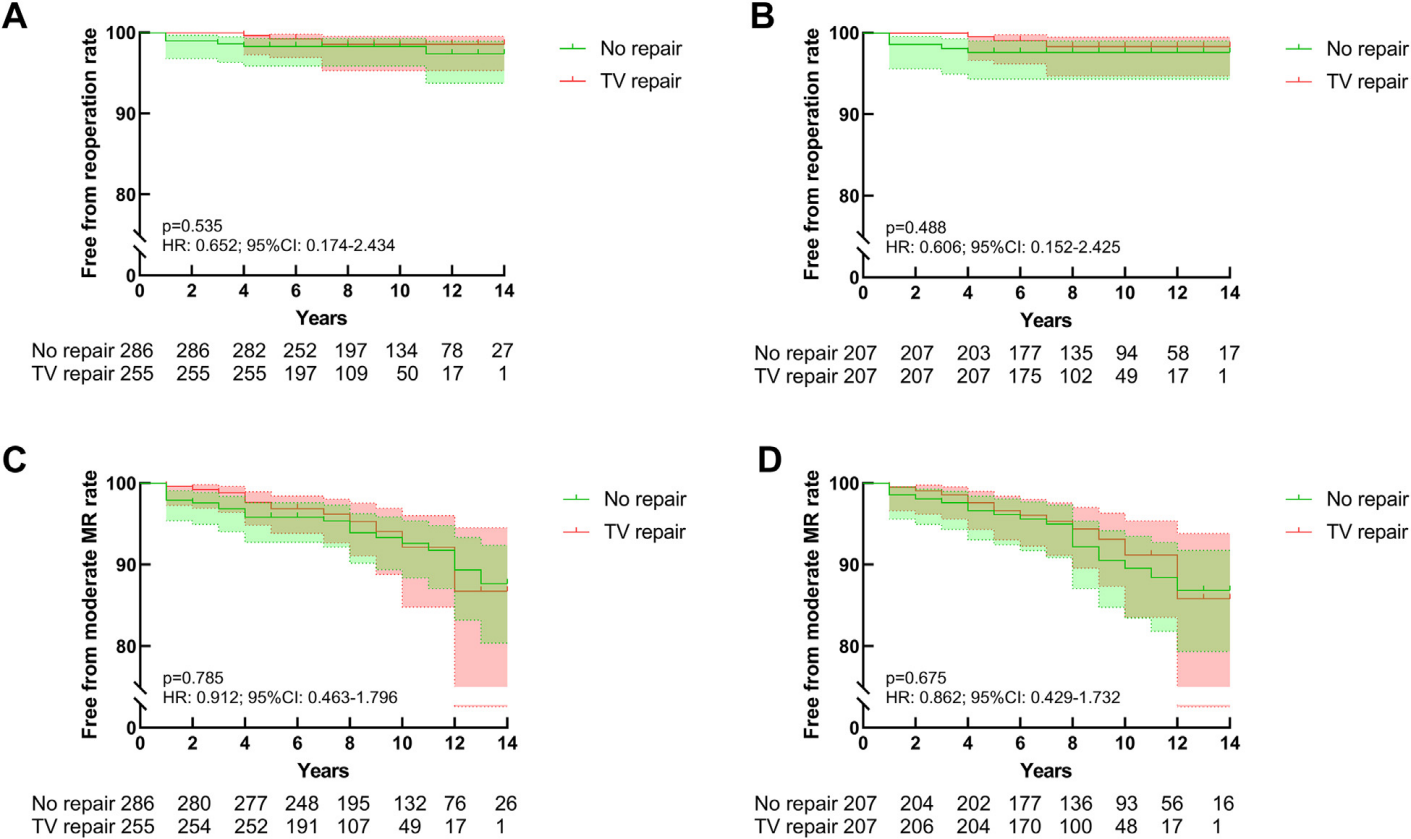
Kaplan-Meier Curves for Reoperation and Recurrent MR (A and B) Cumulative rates of free from the reoperation before and after matching. (C and D) Cumulative rates of freedom from the recurrent mitral regurgitation (MR) before and after matching. TV = tricuspid valve.

Before matching, 9 patients underwent reoperation for MR or TR (3 in the TV-repair group, 6 in the no-repair group), 7 underwent mitral valve replacement, and 2 underwent mitral valve replacement with TV repair. None of the patients had moderate TR before reoperation. Recurrent MR was observed in 35 patients (13 in the TV-repair group and 22 in the nonrepair group). The 2 groups had no significant difference in the reoperation rate (HR: 0.652; 95% CI: 0.174-2.434; *P =* 0.535) and recurrent MR (HR: 0.912; 95% CI: 0.463-1.796; *P =* 0.785). After matching, 8 patients underwent reoperation for MR or TR (3 in the TV-repair group and 5 in the no-repair group), and MR recurred in 32 patients (13 in the TV-repair group and 19 in the no-repair group). Again, there were no significant differences between the 2 groups in the rates of reoperation (HR: 0.606; 95% CI: 0.152-2.425; *P =* 0.488) and recurrent MR (HR: 0.862; 95% CI: 0.429-1.732; *P =* 0.675). Figure 2 shows the before and after matching Kaplan-Meier plots for the cumulative rates of freedom from the development of reoperation and recurrent MR.

### PPM implantation and new-onset atrial fibrillation

**Figure 3:**
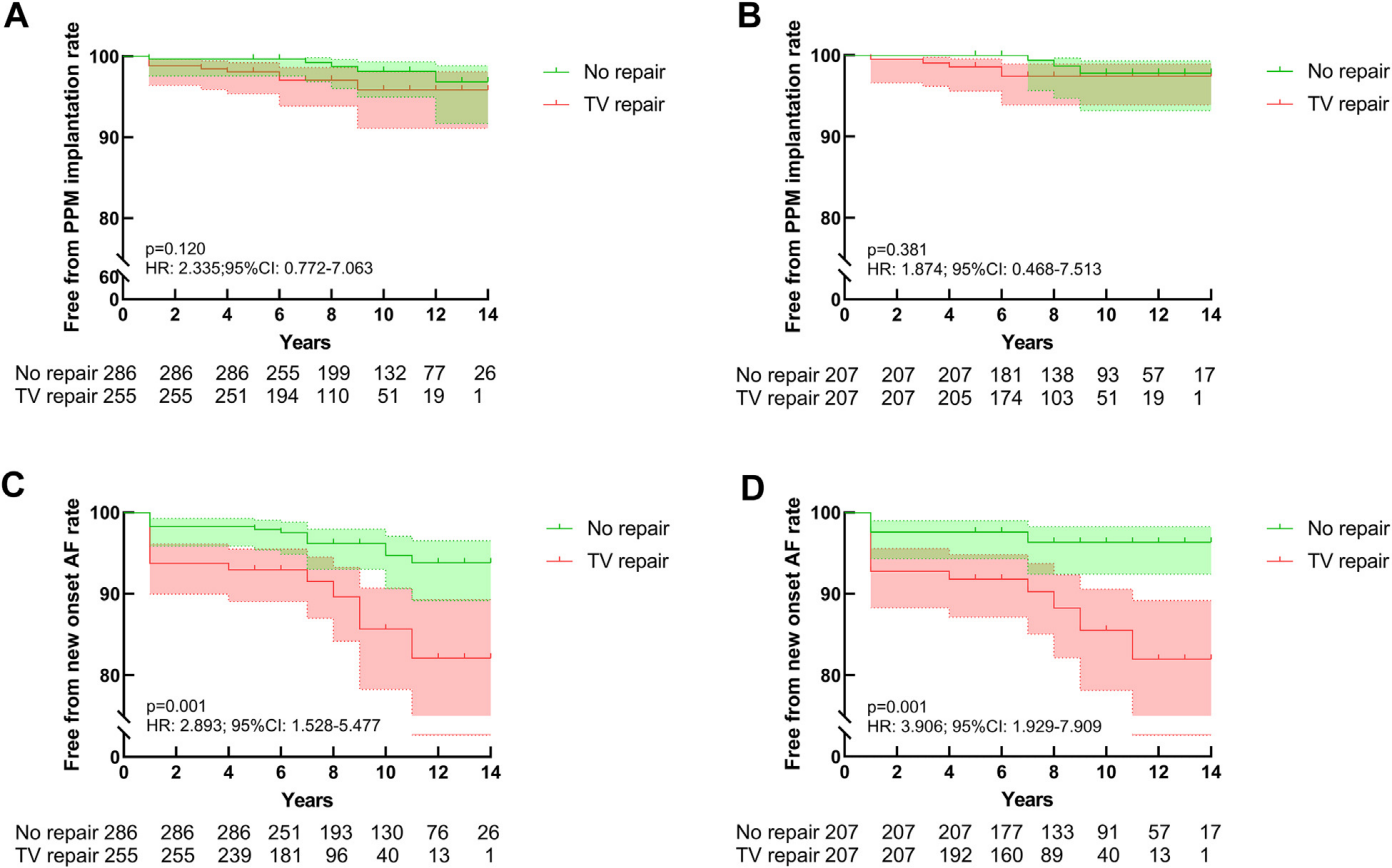
Kaplan-Meier Plots for PPM Implantation and New-Onset Atrial Fibrillation (A and B) Cumulative rates of freedom from the permanent pacemaker (PPM) implantation before and after matching. (C and D) Cumulative rates of freedom from the new-onset atrial fibrillation before and after matching. TV = tricuspid valve.

Thirteen patients received PPM (5 in the TV-repair group and 8 in the no-repair group), and 39 patients developed new-onset atrial fibrillation (31 in the TV-repair group and 8 in the no-repair group) during the follow-up period. There was no significant difference in the rate of PPM implantation between the 2 groups (HR: 2.335; 95% CI: 0.772-7.063; *P =* 0.120). Ten-year freedom from new-onset atrial fibrillation was 84% ± 4% and 94% ± 2% in the TV-repair and no-repair groups, respectively, indicating that the risk of new-onset atrial fibrillation significantly increased with TV repair (HR: 2.893, 95% CI: 1.528-5.477; *P =* 0.001). After matching, 8 patients underwent PPM implantation (5 in the TV-repair group and 3 in the no-repair group), and 31 patients developed new-onset atrial fibrillation (24 in the TV-repair group and 7 in the no-repair group). The results did not change. In fact, additional TV repair increased the risk of new-onset atrial fibrillation (HR: 3.906, 95% CI: 1.929-7.909; *P =* 0.001) but did not increase the risk of PPM implantation. Cox regression analysis showed that only concomitant TV repair was the risk factor for developing new-onset atrial fibrillation (Table 3, Central Illustration). Figure 3 shows Kaplan-Meier plots for the cumulative rates of freedom from PPM implantation and new-onset atrial fibrillation before and after matching.

**Table 3:** Risk Factors for New-Onset Atrial Fibrillation

	HR (95% CI)	*P* Value
LAD	1.03 (0.99-1.06)	0.133
LVEF	1.01 (0.92-1.10)	0.897
LVEDD	0.96 (0.85-1.08)	0.462
LVESD	1.09 (0.91-1.30)	0.354
TV repair	2.74 (1.38-5.44)	0.004

Abbreviations as in Table 1.

**Central Illustration:**
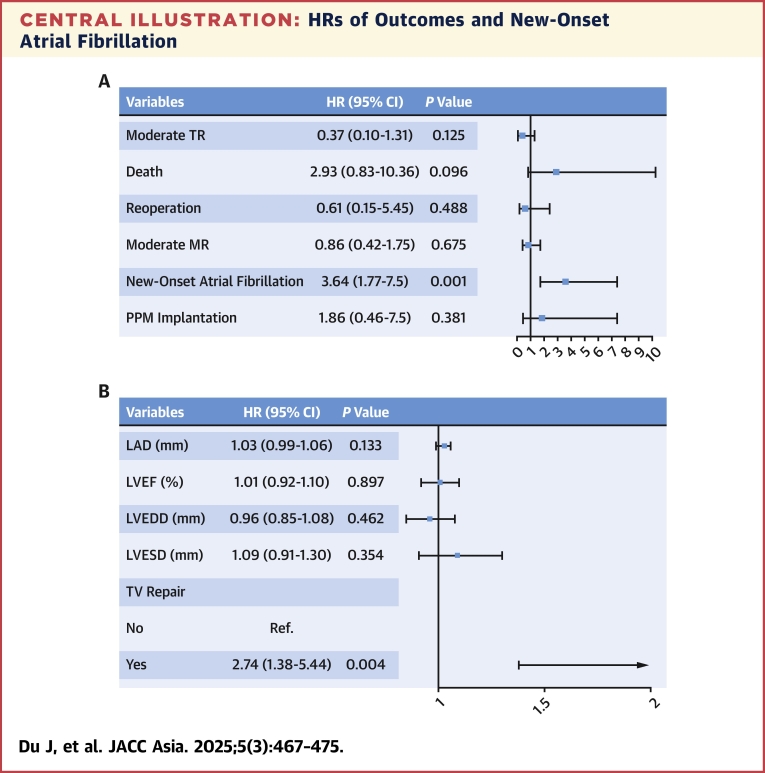
HRs of Outcomes and New-Onset Atrial Fibrillation (A) HR of the primary and secondary outcomes after matching. (B) Multivariate Cox regression analysis of new-onset atrial fibrillation.

## Discussion

In this single-center retrospective analysis, we evaluated the impact of TV repair in patients with less than moderate TR who underwent degenerative mitral surgery. The findings demonstrated that TV repair may not be essential among patients with less than moderate TR, because it had minimal effects on the development of TR during follow-up. However, TV repair among patients with less than moderate TR was associated with a higher incidence of new-onset atrial fibrillation during follow-up.

TR was frequently observed among patients with degenerative MR.^14^ It is widely accepted that severe TR requires surgical treatment, but treating less than severe TR remains controversial. Current practical guidelines offer Class IIa recommendations for TV surgery in patients with progressive TR undergoing left-sided valve surgery who have tricuspid annular dilation or prior signs and symptoms of right-sided HF.^4^ Several studies have also suggested that simultaneous TV repair during mitral surgery in patients with less than severe TR is associated with better outcomes than isolated mitral valve repair.^9^^,^^15^ A prospective randomized cohort study by Gammie et al^6^ revealed that among patients with less than severe TR undergoing mitral valve surgery, the probability of progressing to severe TR was lower in patients after TV repair. These results may explain why aggressive TV repair has recently gained popularity.

However, few clinical studies focused on patients with less than moderate TR. Although previous studies have shown that preoperative moderate TR may be associated with a higher incidence of postoperative mortality,^5^ the postoperative evolution of less than moderate TR may be different. Gammie et al^6^ also reported that the postoperative composite endpoint (including reoperation for TR, progression of TR by 2 grades from baseline, or the presence of severe TR and death) could not be improved by TV annuloplasty among patients with less than moderate TR. In our study, we primarily enrolled patients with less than moderate TR. We found that few patients experienced TR worsening during follow-up. In a study by Kim et al,^16^ they also found that patients with less than moderate TR rarely progressed to severe TR, which aligns with our findings. Based on the previously mentioned findings, we speculate that the progression of less than moderate TR may be relatively stable after isolated mitral surgery, and whether aggressive tricuspid interventions are needed is worth further verification.

Additionally, among these patients, whether concomitant TV repair increases the incidence of postoperative adverse events is still unclear. Although several studies previously reported that concomitant TV repair is associated with a higher risk of PPM implantation,^6^^,^^17^ in this study, we found that additional TV repair was safe when it came to early in-hospital mortality, recurrent MR, reoperation, and PPM implantation, which was in accordance with the results from other similar studies.^9^^,^^15^^,^^18^

As stated earlier, patients with preoperative atrial fibrillation were excluded from this study because we considered the fact that preoperative atrial fibrillation may contribute to the development of TR as has been reported in several previous studies.^16^^,^^19^ Interestingly, we found that the incidence of new-onset atrial fibrillation significantly increased in patients who underwent TV repair, which may be related to the surgical approach. In this study, completely different surgical approaches were used between patients undergoing mitral combined tricuspid surgery and those undergoing isolated mitral surgery. The septal approach was used in the mitral valve combined TV surgery, and some studies have reported that the septal approach is associated with a higher risk of atrial tachycardia when compared to right lateral or left atriotomy,^20^^,^^21^ which is commonly used for isolated mitral surgery. In a study by Markowitz et al,^22^ the septal approach was associated with discrete areas of low voltage within the incision area, including the lateral right atrial wall, anterior to the superior vena cava, the interatrial septum, and the dome of the left atrium, which contribute to the formation of lesional re-entry and tachycardias. Although whether the atrial lesions caused by the septal approach play an important role in the development of new-onset atrial fibrillation deserves more evidence, further prospective and electrophysiological studies are recommended.

### Study limitations

This was a single-center retrospective study and was subject to inherent biases associated with the study design. Additionally, echocardiography and ECG data could not be continuously obtained during follow-up, and some patients were not regularly re-examined, thereby reducing the accuracy of the occurrence time of endpoints.

## Conclusions

Considering its minimal effects on the development of TR and potential contributions to postoperative new-onset atrial fibrillation during follow-up among these patients, more aggressive TV repair is not encouraged among patients with less than moderate TR during MV surgery.

## Funding Support and Author Disclosures

The authors have reported that they have no relationships relevant to the contents of this paper to disclose.
